# ﻿*Carexduanensis* (Carexsect.Rhomboidales), a new species of Cyperaceae from limestone areas of Guangxi, China

**DOI:** 10.3897/phytokeys.241.121098

**Published:** 2024-05-02

**Authors:** Zhao-Cen Lu, Yi-Fei Lu, Shi-Li Chang, Ming-Lin Mo, Xiao-Feng Jin

**Affiliations:** 1 Guangxi Key Laboratory of Plant Conservation and Restoration Ecology in Karst Terrain, Guangxi Institute of Botany, Guangxi Zhuang Autonomous Region and Chinese Academy of Sciences, Guilin, 541006, Guangxi, China Guangxi Institute of Botany Guilin China; 2 School of Forestry and Biotechnology, Zhejiang A&F University, Hangzhou, 311300, Zhejiang, China Zhejiang A&F University Hangzhou China; 3 College of Life Sciences, Guangxi Normal University, Guilin, 541006, Guangxi, China Guangxi Normal University Guilin China

**Keywords:** *Carexduanensis*, Cyperaceae, morphology and micromorphology, nutlet, taxonomy, utricle

## Abstract

*Carexduanensis* Z.C.Lu, Y.F.Lu & X.F.Jin, a new species in limestone areas of Guangxi, China, was discovered and described. The morphology showed that *C.duanensis* is similar to *C.calcicola*, but differs in having culms central, leaf blades 3–5.5 mm wide, bracts longer than spikes, utricles 4–5 mm long, shorter, and nutlets abruptly contracted into an erect beak at apex. SEM microphotographs of utricles and nutlets are provided for the new and related species, *C.calcicola*.

## ﻿Introduction

Cyperaceae (sedges), containing 5600+ species in 95 genera worldwide, are the third largest monocot family. It is distributed from tropical to Polar regions, from alpine meadows to tropical rainforests, from Gobi deserts to swampy wetlands, and plays a crucial role in various ecosystems (Larridon et al. 2021). The genus *Carex* L., comprising ca. 2000 species, stands as the largest genus within Cyperaceae and is distributed almost worldwide (Reznicek 1990; Starr and Ford 2009; Roalson et al. 2021). Recent phylogenetic studies have provided a framework for identifying morphologically diagnosable lineages within *Carex*, organizing it into six subgenera: Carexsubg.Siderosticta, C.subg.Psyllophorae, C.subg.Euthyceras, C.subg.Uncinia, C.subg.Vignea, and C.subg.Carex. Moreover, the genus was subdivided into 62 formally named Linnean sections plus 49 informal groups (Roalson et al. 2021). *Carex* is one of the largest genera of angiosperms in China, boasting representation of more than 700 species (Chen and Zhang 2018).

CarexsectionRhomboidales was established by Kükenthal in his worldwide monograph of *Carex* (Kükenthal 1909). Jin and Zheng (2013) revised this section and recognized 40 species, along with six subspecies and four varieties. Since then, some new species of C.sect.Rhomboidales have been described and published (Chen and Jin 2015; Yang et al. 2015a, 2015b, 2016). Recent phylogenetic studies revealed that C.sect.Rhomboidales is monophyletic (Roalson et al. 2021), comprising 50+ species mainly distributed in East and Southeast Asia, with a few species occurring from Europe to western Asia (Kükenthal 1909; Dai et al. 2000, 2010; Jin and Zheng 2013; Roalson et al. 2021).

During the investigation from May 2021 to March 2023, we collected specimens of *Carex* with mature nutlets from limestone evergreen broad-leaved forests and shrubs in Du’an County and Yizhou City, Guangxi, China. After carefully checking the morphological characters of these specimens and comprehensively consulting relevant literature, with comparison of nutlet and utricle micromorphology (Dai et al. 2000, 2010; Jin and Zheng 2013; Jin et al. 2014; Chen and Jin 2015; Yang et al. 2015a, 2015b, 2016; Roalson et al. 2021), we confirmed it as a new species of C.sect.Rhomboidales and described below.

## ﻿Materials and methods

Specimens of this new species were collected from Du’an County and Yizhou City, Guangxi, China. After that, we carefully studied relevant literature and the morphological characters of the specimens, which involved measuring and recording the size, shape, and color of rhizomes, culms, leaves, bracts, spikes, glumes, utricles, and nutlets. We examined herbarium specimens at BM, E, HTC, IBK, IBSC, K, KUN, P, PE and ZJFC. The other related species of Carexsect.Rhomboidales were examined online images from Kew Herbarium Catalogue (http://apps.kew.org/herbcat/gotoHomePage.do), JSTOR Global Plants (https://plants.jstor.org/) and Chinese Virtual Herbarium (https://www.cvh.ac.cn/).

SEM (scanning electron microscope) observations of utricles and nutlets of the new species and the similar species *Carexcalcicola* Tang & F.T.Wang in C.sect.Rhomboidales were carried out. Mature utricles and nutlets were gathered from specimens we collected, the specimens ‘*W. B. Xu, C. R. Lin & Z. C. Lu 14641*’ for the new species and ‘*X. F. Jin & al. 2391*’ for *C.calcicola* respectively. The utricles were submerged in 50% ethanol to clean for 2 hours, then air dried. The cleaned utricles were mounted on stubs by doubled-sided adhesive tape and coated with gold. The nutlets were initially soaked in a solution of concentrated sulfuric acid and acetic anhydride (volume ratio = 1:9) for 16 hours, then rinsed in acetic acid for 10 min and water for 5 min, and placed in a bath-type ultrasonic cleaner for 30 min with 70% ethanol to remove the cuticle and outer periclinal wall of the epidermis (Jin et al. 2014). After air drying, the nutlets were also mounted on stubs using double-sided adhesive tape, and directly coated with a layer of gold. The coated utricles and nutlets were observed and photographed under a GEMINI-300 scanning electron microscope (SEM).

## ﻿Taxonomic treatment

### ﻿ Carex duanensis

Taxon classificationPlantaePoalesCyperaceae

Z.C.Lu, Y.F.Lu & X.F.Jin sp. nov.

0893C672-9830-5648-BA15-4FB81BDCED87

urn:lsid:ipni.org:names:77340986-1

**Figure 1.**
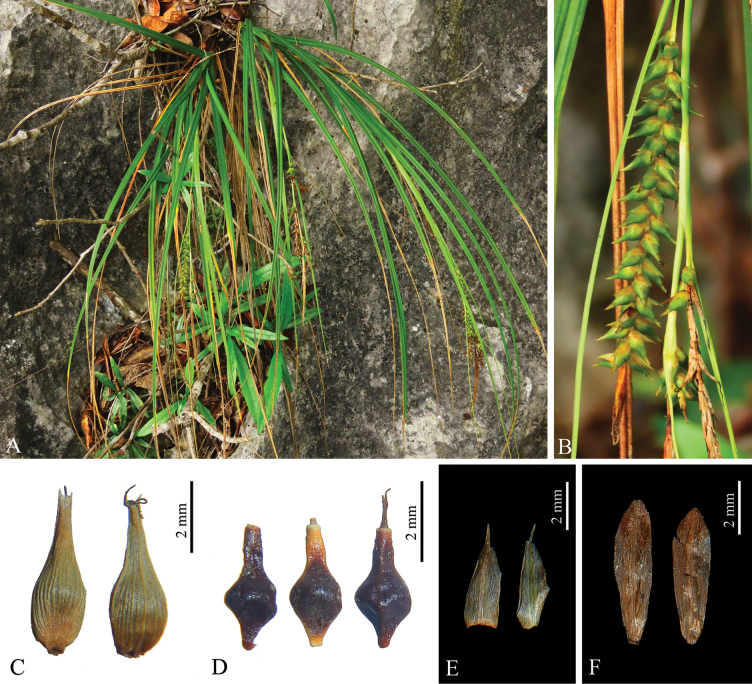
*Carexduanensis* sp. nov. **A** habit **B** lateral spikes **C** utricles **D** nutlets **E** pistillate glumes of middle and upper part **F** staminate glumes.

**Figure 2.**
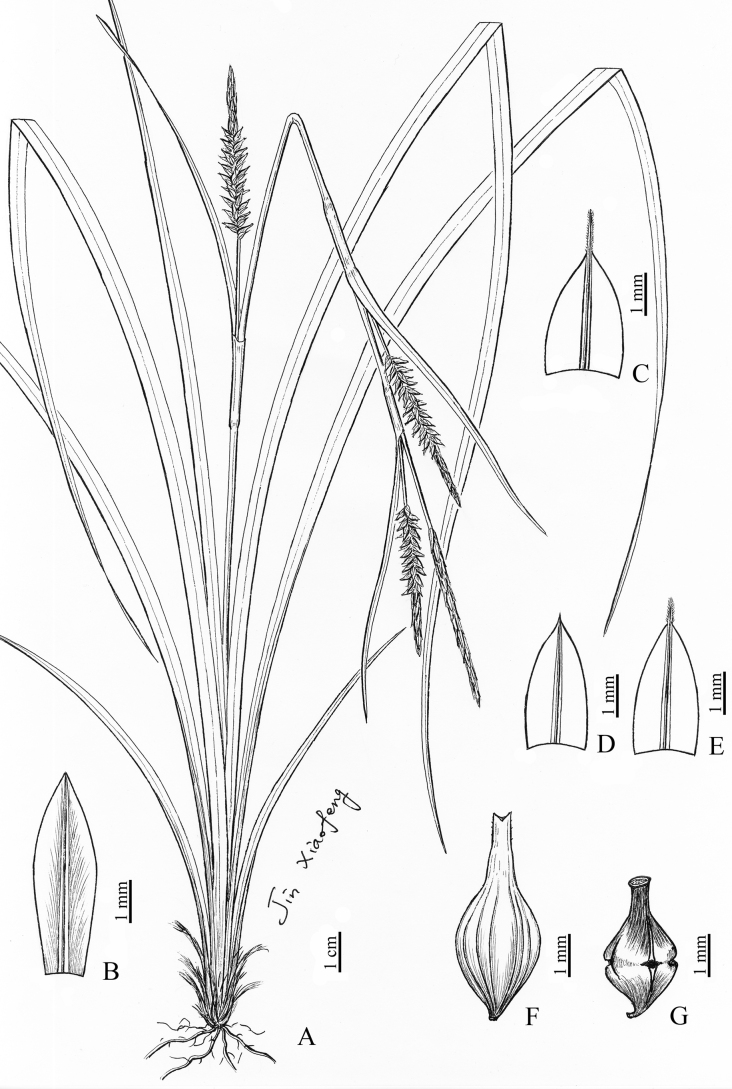
*Carexduanensis* sp. nov. **A** habit **B** staminate glume **C** pistillate glume of basal part **D, E** pistillate glumes of middle and upper part **F** utricle **G** nutlet. (Drawn by Xiao-Feng Jin; based on the holotype: *W.B.Xu et al. 14641* in IBK).

Figs 1A–F , 2A–G Chinese name: dū ān tái cǎo (都安薹草)

#### Diagnostic description.

This new species is similar to *Carexcalcicola* Tang & F.T.Wang, but differs in having culms central (vs. culms lateral), leaf blades 3–5.5 mm wide (vs. 7–15 mm wide), bracts longer than spikes (vs. shorter than spikes or nearly equal in length), utricles 4–5 mm long (vs. 5.5–6.5 mm long), shorter, and nutlets abruptly contracted into an erect beak at apex (vs. curved or coiled beak at apex).

#### Type.

China. Guangxi: Du’an County, National Geopark in Dongmiao Town, 23°59'41"N, 107°59'14"E, limestone slope, alt. 240 m, 5 May 2021, *W. B. Xu, C. R. Lin & Z. C. Lu 14641* (holotype: IBK! barcode IBK00457898; isotypes: IBK! barcode IBK00457899, ZJFC!).

#### Description.

Perennial herbs. Rhizomes short, woody, thick. Culms central, caespitose, 20–50 cm tall, trigonous, slightly scabrous, base with dark brown fibrous sheaths. Leaves longer than culms; blades 3–5.5 mm wide, leathery, margin slightly involuted, scabrous on margins and abaxial leaf surfaces. Bracts leaf-like, longer than spikes, sheathed; sheaths 10–30 mm long. Spikes 3–6; terminal spike staminate, clavate-cylindrical, 3–7 cm long, 2–3 mm wide, base with a 1.5–2.5 cm long peduncle; lateral spikes androgynous, rarely pistillate, cylindrical, 3–5(–8) cm long, 5–7 mm wide, staminate part 1–3 cm long, densely flowered, pistillate part 1.5–4 cm long, sparsely flowered, base with a 1–5 cm long peduncle; peduncles enclosed or slightly exserted from bract sheaths. Staminate glumes oblong-lanceolate, 4–5.5 mm long, yellow-brown, obtuse or acuminate at apex, pale yellow-brown 3-veined dorsal costa. Pistillate glumes (basal part) broadly ovate, ca. 4 mm long, yellow-white, acuminate at apex, yellow 3-veined dorsal costa excurrent into a scabrous awn, middle and upper ones ovate, 3–3.5 mm long, yellow-white, acuminate at apex, yellow 3-veined dorsal costa excurrent into a 0.5–1 mm long scabrous awn. Utricles yellow-green, ovoid (excluding beak), obtusely trigonous, 4–5 mm long (including beak), longer than pistillate glumes, chartaceous, distinctly thinly veined, glabrous, base cuneate, apex gradually contracted into a 2–2.2 mm long beak, orifice 2-lobed with short teeth. Nutlets tightly enveloped, rhombic-ovoid, trigonous, castaneous, 2.5–3 mm long, with 3 angles constricted at middle, sides concave above and below, base with a curved stipe, apex abruptly contracted into a ca. 1 mm long beak, beak erect or slightly curved, annulate at orifice; style thickened at base; stigmas 3.

#### Etymology.

The specific epithet ‘*duanensis*’ refers to the type locality of this new species.

#### Phenology.

Flowering and fruiting mid-March to early May.

#### Distribution and Habitat.

*Carexduanensis* has only been collected from limestone areas of Guangxi, China. It is currently known to grow sporadically in forests or shrubs on limestone slopes, at an elevation of 200–500 m.

#### Additional specimens examined

**(paratypes).** China. Guangxi: Du’an County, National Geopark in Dongmiao Town, 23°59'41"N, 107°59'14"E, limestone slope, alt. 240 m, 5 May 2021, *W. B. Xu, C. R. Lin & Z. C. Lu 14640* (IBK!, ZJFC!); the same locality, 5 May 2021, *W. B. Xu, C. R. Lin & Z. C. Lu 14642* (IBK!, ZJFC!); Nongshui, Yu’an Village of Dongmiao Town, 23°57'47.37"N, 107°56'39.37"E, in limestone thickets slope, alt. 290 m, 13 March 2023, *W. B. Xu, C. R. Lin, Z. C. Lu & J. Q. Huang 15556* (IBK!); Nongyi, Ditong Village of Dongmiao Town, 23°58'51.52"N, 107°52'06.17"E, in limestone slope, alt. 465 m, 13 March 2023, *W. B. Xu, C. R. Lin, Z. C. Lu & J. Q. Huang 15579* (IBK!); Yizhou City, Latan Village of Anma Town, 24°42'14"N, 108°28'07"E, under limestone forests, alt. 250 m, 1 May 2021, *W. B. Xu, C. R. Lin & Z. C. Lu 14459* (IBK!).

#### Conservation status.

The new species has been found in three localities in Du’an County and one locality in Yizhou City, Guangxi, China. One locality is in Du’an County, National Geopark in Dongmiao Town, two localities are in the assessment area of Southwest Karst National Park, which is currently being prepared; the populations are in protected areas where they are less threatened. According to the IUCN Red List Categories and Criteria (IUCN 2022), *Carexduanensis* will be considered in the Least Concern (LC) category.

#### SEM micromorphology.

**Figure 3.**
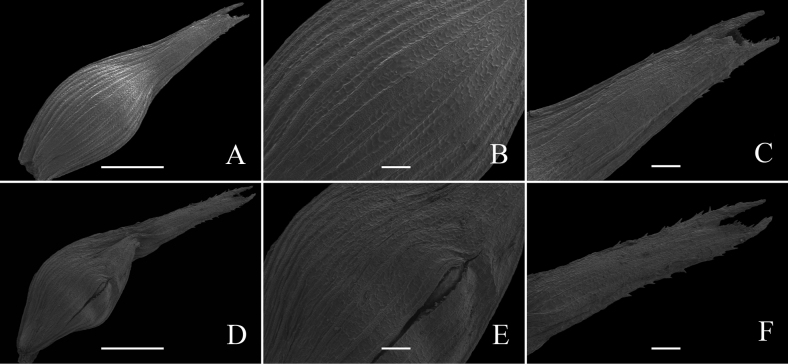
SEM micromorphology of utricles of *Carexduanensis* (**A–C**) and *C.calcicola* (**D–F**) **A, D** overview **B, E** surface **C, F** beak . Scale bars: 1 mm (**A, D**); 200 μm (**B, C, E, F**)

**Figure 4.**
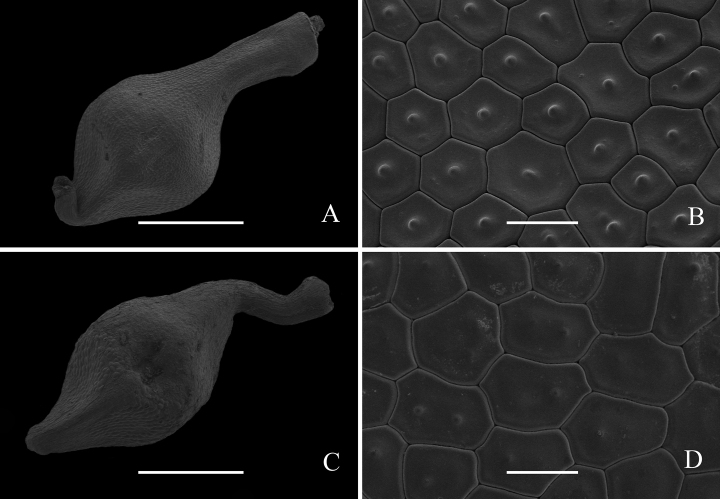
SEM micromorphology of nutlets of *Carexduanensis* (**A, B**) and *C.calcicola* (**C, D**) **A, C** overview **B, D** surface, Scale bar: 1 mm (**A, C**); 50 μm (**B, D**)

SEM micromorphology uses detailed descriptions which are shown in Figs 1, 2. The utricles of *Carexduanensis* and *C.calcicola* are both obovoid, glabrous, with many longitudinal veins; beak margins prickly-hairy, and short and sharp 2-teethed at the top (Fig. 3), but the utricle length of *C.duanensis* is slightly shorter than those of *C.calcicola*. The nutlet shapes of *C.duanensis* and *C.calcicola* are both rhombic-ovoid, with 3 angles constricted at middle and epidermal cells 5- or 6-gonal, rarely 4-gonal, with straight anticlinal walls and solitary rarely 2 silica bodies. The nutlet beak of *C.duanensis* is erect, whereas that of *C.calcicola* is curved (Fig. 4).

#### Notes.

*Carexduanensis* has nutlets rhombic-ovoid, obtusely trigonous, with 3 angles constricted at middle, sides concave above and below, apex abruptly contracted into a ca. 1 mm long beak, beak erect or slightly curved, annulate at orifice. Based on these morphological characters, *C.duanensis* belongs to C.sect.Rhomboidales and is similar to *C.calcicola* (Dai et al. 2010), but differs from the latter in having culms central, leaf blades 3–5.5 mm wide, bracts longer than spikes, utricles 4–5 mm long, shorter, and nutlets abruptly contracted into an erect beak at apex. The morphological differences of *C.duanensis* and *C.calcicola* are shown in Table 1.

**Table 1.** Morphological characters distinguishing *C.duanensis* from *C.calcicola*.

Characters	*C.duanensis*	*C.calcicola*
1. Culms	Central	Lateral
2. Leaf blades	3–5.5 mm wide	8–15 mm wide
3. Bracts	Longer than spikes	Shorter than spikes
4. Utricles	4–5 mm long	5.5–6.5 mm long
5. Nutlets	Abruptly contracted into an erect or slightly curved beak at apex	Abruptly contracted into a coiled or curved beak at apex

## Supplementary Material

XML Treatment for Carex duanensis

## ﻿Additional information

### Author ORCIDs

Zhao-Cen Lu https://orcid.org/0000-0002-1725-0247

Yi-Fei Lu https://orcid.org/0000-0001-6095-3821

Shi-Li Chang https://orcid.org/0009-0005-9801-4121

Ming-Lin Mo https://orcid.org/0009-0000-8895-5474

Xiao-Feng Jin https://orcid.org/0000-0002-8195-6738

### Data availability

All of the data that support the findings of this study are available in the main text.
